# Sexual minority status, social adversity and risk for psychotic disorders-results from the GROUP study

**DOI:** 10.1017/S0033291719003726

**Published:** 2021-04

**Authors:** D. Post, W. Veling, GROUP investigators

**Affiliations:** Department of Psychiatry, University of Groningen, University Medical Center Groningen, Groningen, The Netherlands

**Keywords:** LBG, psychosis, sexual minority stress, social defeat

## Abstract

**Background:**

Lesbian, bisexual, or gay individuals (LBGs) have an increased risk for mental health problems compared to heterosexuals, but this association has sparsely been investigated for psychotic disorders. The aim of this study was: (1) to examine whether LBG sexual orientation is more prevalent in individuals with a non-affective psychotic disorder (NAPD) than in people without a psychotic disorder; and if so, (2) to explore possible mediating pathways.

**Methods:**

Sexual orientation was assessed in the 6-year follow-up assessment of the Dutch Genetic Risk and Outcome of Psychosis study (GROUP), a case–control study with 1547 participants (582 patients with psychotic disorder, 604 siblings, and 361 controls). Binary logistic regression analyses were used to calculate the risk of patients with a psychotic disorder being LBG, compared to siblings and controls. Perceived discrimination, history of bullying, childhood trauma (CT), and sexual identity disclosure were investigated as potential mediating variables.

**Results:**

The proportion of individuals with LBG orientation was 6.8% in patients (*n* = 40), 4.3% in siblings (*n* = 26), and 2.5% in controls (*n* = 10). The age- and gender-adjusted odds ratio of LBG for patients was 1.57 (95% CI 1.08–2.27; *p* = 0.019), compared to siblings and controls. Discrimination, bullying, and CT all partially mediated this association.

**Conclusions:**

Adverse social experiences related to sexual minority status may increase the risk for NAPD. Sexual identity, behavior, and difficulties need more attention in everyday clinical practice.

## Introduction

During the late 1950s, when homosexuality was still viewed as a psychiatric disorder, non-clinical population-based studies in the *visible* lesbian, bisexual, or gay individual (LBG) community repeatedly found no elevation of the natural occurrence of mental disorders in LBGs compared to heterosexual (HTS) people (Cochran & Mays, 2000). Since the early 1990s however, research with improved study designs and less selective inclusion of LBG individuals reported increased rates of mental disorders in LBGs compared to HTSs. A meta-analysis of 25 studies calculated odds ratios (ORs) of 1.5 for depression, anxiety, and substance abuse disorders, and a twofold excess in suicide attempts (King et al., 2008). In a large majority of studies, however, psychosis was not investigated as a mental health outcome. Sexual minority status has been associated with a higher prevalence of psychotic symptoms in general population studies in the UK and the Netherlands (Chakraborty, McManus, Brugha, Bebbington, & King, 2011; Gevonden et al., 2014). To the best of our knowledge, these are the only two studies comparing risk for psychotic disorders and psychotic symptoms between LBGs and HTSs, respectively. The current study aimed to investigate the association between LBG status and the risk for psychotic disorders, and to explore potential pathways.

Social adversity and social stress over the life course may be a substantial mediator of psychological problems and mental illness in LBGs. Social stress occurs when the social self is threatened due to maltreatment, stigmatization, discrimination, or exclusion (Meyer, 2003). Such social-evaluative threats are more likely to occur for those belonging to ethnic (Veling, 2013) and sexual (Kuyper & Fokkema, 2011) minority groups and may increase the risk for psychiatric disorders. The prevalence of childhood sexual and physical abuse is up to four times more likely to occur in LBGs (Corliss, Cochran, & Mays, 2002). Gay boys are 4.6 times, and lesbian girls are 2.4 times more likely to be bullied during high school compared to HTS adolescents (Goodenow, Watson, Adjei, Homma, & Saewyc, 2016). There is tentative evidence for a dose–response relationship between victimization through bullying and mental health problems (Bontempo & D'Augelli, 2002). Moreover, childhood bullying is specifically thought by some to influence cognitive and biological mechanisms of psychotic ideation in those at-risk mental states in early adolescence (Lataster et al., 2006).

**Fig. 1.**
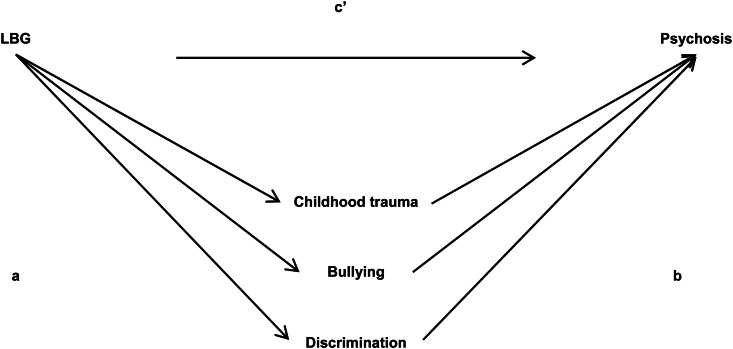
Sexual minority stress and NAPD. Arrows marked with letters (a, b, c’) represent the different parameters to be tested in a mediation analysis.

To our knowledge, associations between sexual minority status and psychotic disorders have not been studied (see Fig. 1). A fair amount, however, has been published on the socially adverse environmental risk factors for non-affective psychotic disorder (NAPD). The association between childhood trauma (CT) and psychosis has been quantified to a substantial OR of 2.8 (van Nierop et al., 2014). Childhood bullying increases the risk for psychotic mental disease (Bebbington et al., 2004). Lastly, perceived discrimination too has been associated with an increased risk of psychotic symptoms in clinical minority studies (Pearce, Rafiq, Simpson, & Varese, 2019). Within LBG populations, the degree of perceived discrimination by means of sexual prejudice has been associated with mental health problems (Goodenow et al., 2016).

Factors of social adversity thought to mediate associations between LBG and psychosis are shown in Fig. 1. A previous cross-sectional study (Gevonden et al., 2014) found that perceived discrimination, in particular, mediated the twofold increased psychotic symptom development in a community sample of LBGs.

The current study investigated the prevalence of LBG in a large population-based cohort of patients with psychotic disorders, their siblings, and healthy controls. We aimed: (1) to examine whether the proportion of LBGs is higher in patients with psychotic disorders compared to individuals without psychotic disorder; and if so, (2) to explore possible mediating pathways. We hypothesized: (a) that sexual minority status is more common in patients than in siblings and healthy controls, (b) that patients less often disclose their sexual identity to others, and (c) that CT, experiences of bullying, and perceived discrimination contribute to an increased risk for NAPD.

## Methods

Data were collected from the Genetic Risk and Outcome in Psychosis Study (GROUP) (Korver, Quee, Boos, Simons, & de Haan, 2012), a large longitudinal observational population-based cohort study, conducted in Dutch mental health institutes affiliated with four academic medical centers in the Netherlands (Amsterdam, Groningen, Maastricht, Utrecht) and in regional psychotic disorder services in Belgium. The procedure of recruitment and population characteristics has been described in detail elsewhere (Korver et al., 2012). The GROUP-study was approved by the Medical Ethics Committee of the Academic Medical Center of Utrecht. All subjects gave written informed consent. The current study uses data from the third GROUP assessment, 6 years after baseline (data assessment 2011–2014).

### Subjects

Patients were asked to participate in the GROUP study if they met the following inclusion criteria: (i) age range 16–50 years, (ii) diagnosis of (recent) NAPD, and (iii) good command of the Dutch language. Control subjects were selected through a system of random mailings to addresses in corresponding geographical areas. Controls were excluded if they had a first-degree relative with a psychotic disorder, established with the Family Interview for Genetic Studies. Siblings of included patients were also approached to take part in the GROUP study, if they did not have a history of psychotic disorder. If controls or relatives developed a psychosis during the study period, they were allocated to the patient group.

### Measurements

#### Diagnostic instruments

Detailed medical and psychiatric histories were collected, including the Comprehensive Assessment of Symptoms and History (CASH), a semi-structured interview for assessing diagnosis and psychopathology (Andreasen, Flaum, & Arndt, 1992); or Schedules for Clinical Assessment for Neuropsychiatry (SCAN 2.1) (Wing et al., 1990). Trained psychologists or psychiatrist with extensive clinical experience using the Diagnostic and Statistical Manual of Mental Disorders-IV (DSM-IV) criteria (APA, 2000) made diagnostic classification(s).

#### Sexual orientation and behavior

Homosexuality has several dimensions, including self-identification, same-sex attraction, and same-sex behavior. In order to capture the best dimension of sexual orientation, participants were asked if their predominant orientation was same-sex (response options ‘yes’, ‘no’, ‘I don't know’, and ‘refuse to answer’). Participants were classified as LBG if they replied ‘yes’. Missing data for sexual minority status, i.e. ‘I don't know’ or ‘refuse to answer’, were recoded as ‘no’ and assigned subjects to the HTS group. In a sensitivity analysis, we recoded ‘I don't know’ and ‘refuse to answer’ into ‘yes’ in order to compare both results. All participants were also asked to what extent they had disclosed their sexual orientation to people in their environment. Sexual identity disclosure can be seen as weakening effect modifier of (minority) stress (Kuyper & Fokkema, 2011). The latter was illustrated by findings of lower cortisol levels and less psychiatric symptoms in adult LGBs who had disclosed their sexual identity compared to those who had not (Juster, Smith, Ouellet, Sindi, & Lupien, 2013). Disclosure is also associated with affiliation and formation of social circuits, which are likely to reduce the impact of social stress (Meyer, 2003).

#### Socio-demographic variables

Socio-demographic variables included age, gender, ethnicity (% of Caucasian participants), living with a partner, education (% highest degree obtained), urban living (see Table 1), and lifetime cannabis use (% of participants that ever used cannabis during their lifetime ‘yes/no’).

**Table 1.** Socio-demographic and clinical characteristics of the study sample

Sexual identity status	LBG (*N* = 76)			HTS (*N* = 1470)		
*N*	Patients 40	Siblings 26	Controls 10	Patients 542	Siblings 578	Controls 351
Male (%)	29 (72.5)	14 (53.8)	6 (60.0)	408 (75.3)	261 (45.0)	155 (44.2)
Age mean (s.d.)	33.7 (7.4)	34.1 (8.2)	37.5 (10.6)	34.9 (8.7)	34.9 (8.7)	35.2 (8.8)
Ethnicity white (%)	34 (89.5)	21 (84.0)	10 (100.0)	440 (83.8)	504 (89.5)	319 (93.3)
Married/living together (%)	8 (20.0)	10 (38.5)	4 (40.0)	113 (20.8)	384 (66.6)	221 (67.0)
High level of education^a^ (%)	14 (35.0)	12 (46.2)	8 (80.0)	131 (24.2)	310 (53.6)	222 (63.2)
Urban living mean^b^	9.0	8.1	14.0	6.9	6.6	6.7
Psychotic disorder (%)	100.0	0.0	0.0	100.0	0.0	0.0
Mood disorder (%)	5.3	29.2	30.0	4.0	20.5	16.6
Anxiety (%)	–	–	–	0.2	0.4	0.6
Personality disorder (%)	0.0	4.2	0.0	0.0	0.4	0.3
Miscellaneous (%)	2.6	66.7	70.0	0.8	78.6	82.5
Cannabis lifetime ever used (yes/no) (%)^c^	17.5	38.5	20.0	25.6	13.6	10.8
Childhood Trauma Questionnaire mean^c^	1.6	1.8	1.4	1.5	1.4	1.3
Bullied mean^c^	2.4	1.9	1.6	1.8	1.5	1.3
Discrimination mean^c^	2.4	0.9	1.0	1.5	0.7	0.7

a Higher vocational education or university degree.

b Urban living was computed by sum scores (maximum score of 15) to reflect the amount of times participants lived in a densely populated area (i.e. >1000 to >2500 persons/km^2^) between the age of 0 and 19; addresses were coupled to the national database of Statistics of The Netherlands.

c Percentage of missing data: ethnicity 13.5%, urban living 76.1%, Cannabis Lifetime ever used 4.3%, CTQ-SF 12.3%, bullied 4.4%, discrimination 10.5%.

#### Social adversity and social stress

CT was assessed with the Dutch version of the Childhood Trauma Questionnaire-Short Form (CTQ-SF). The Dutch CTQ-SF effectively screens for maltreatment between clinical and non-clinical samples (Thombs, Bernstein, Lobbestael, & Arntz, 2009). The CTQ-SF is a 25-item retrospective self-report questionnaire designed to assess five dimensions of childhood maltreatment: (1) Physical Abuse, (2) Emotional Abuse, (3) Sexual Abuse, (4) Physical Neglect, and (5) Emotional Neglect. The total mean score of all child trauma experiences was used for analysis.

Bullying was assessed as follows: participants were asked if they had ever been bullied by another child or teenager during elementary, middle-, or high school and asked to rate the severity of bullying on a five-point scale (from never **=** 1 to often **=** 5). Lifetime discrimination experiences were assessed with a series of dichotomous ‘yes’ or ‘no’ questions on the following situations: ever been fired, not hired for a job, not been promoted, detained, questioned or threatened by police, badly treated by the justice system, discouraged from further education, prevented to buy/let a house, badly treated by neighbors, denied a loan/mortgage, received bad service, or been badly treated in either medical care or public transport. The mean cumulative score was used as a measure of perceived discrimination. In contrast to CT, which was assessed at wave 2, bullying, discrimination, and sexual minority were all assessed at wave 3.

### Statistical analysis

Statistical analysis was performed using SPSS 17.0. Pearson χ^2^ test of independence, independent samples *t* test, and ANOVA (one-way) were used to test socio-demographic and clinical differences between patients and controls, and between LBG and HTS groups.

Binary logistic regression analyses were used to compare the risk (expressed as OR) of patients with a psychotic disorder being LBG, compared to people without a psychotic disorder. *A priori* determined confounding variables of age and gender were adjusted for.

To investigate whether CT, bullying, and perceived discrimination mediated the association between LBG and NAPD, a bootstrapped multiple mediation analysis was conducted with the Process macro developed by Hayes (2012). Release 6.0 of the GROUP database was used for analyses.

## Results

A total of 1766 participants were assessed at wave 3 of the GROUP study. Information was available for 1546 patients (87.5%) of which, 582 patients, 603 siblings, and 361 controls. Analyses were based on these 1546 subjects. As depicted in Table 1, 6.8% of patients (40 out of 582), 4.3% (26 out of 605) of siblings, and 2.5% (10 out of 361) controls were classified as LBGs.

Controls were significantly more likely to be living/married with someone than patients (OR 3.2, 95% CI 2.7–3.8). The proportion of people with high education was lower in patients than in controls. LBG patients had significantly higher mean scores for CT when compared to HTS patients. In the LBG group, 39.5% (*n* = 15) of patients reported often bullying *v.* 20% (*n* = 2) in the control group. In HTS participants, this was 20.3% and 8.1%, respectively. Discrimination scores were also significantly higher in the LBG participants, with 29% of patients and 50% of controls reported never to have experienced discrimination *v.* 39% and 62% in HTS counterparts.

Data of all 1546 subjects were used to calculate binary regression estimates (see Table 2). Compared to controls, the OR of LBG status was 1.61 for patients with NAPD (95% CI 1.13–2.29–4.92, *p* = 0.008) and for siblings was 1.58 (95% CI 0.75–3.32, *p* = 0.225). Of the LBG participants: 78% of controls, 38% of siblings, and 29% of patients had disclosed their sexual orientation to almost everyone in their lives and not a single control, 4% of siblings, and 16% of patients reported that no one knew of their sexual orientation.

**Table 2.** Sexual minority status and risk for psychotic disorders

Sexual minority status	Unadjusted			Adjusted^a^		
	OR	95% CI	*p* value	OR	95% CI	*p* value
Psychotic disorders	1.61	1.13–2.29	0.008	1.57	1.08–2.27	0.019
Siblings	1.58	0.75–3.32	0.225	1.58	0.74–3.37	0.235
Healthy controls	1.0	–	–	1.0	–	–

a Adjusted for age and gender.

Multiple mediation analysis showed (see Table 3) that perceived discrimination, CT, and bullying all partially mediated the association between LBG status and NAPD. The indirect effect of discrimination, controlling for the other mediators, was the greatest in predicting psychosis in LBGs, *B* = 0.23 (bootstrapped 95% CI 0.07–0.44). The second greatest indirect effect was that of CT, *B* = 0.12 (bootstrapped 95% CI 0.04–0.25) and the last was bullying, *B* = 0.06 (95% CI 0.002–0.15).

**Table 3.** Multiple mediation analysis of relationship between LBG status and psychotic disorder

Indirect effect	*B*	95% CI
Total	0.40	0.19–0.65*
Childhood trauma	0.12	0.04–0.25*
Bullied in adolescence	0.06	0.002–0.15*
Lifetime discrimination	0.23	0.07–0.44*

Mediation analysis using Hayes' PROCESS macro in SPSS.

* *p* < 0.05.

In sensitivity analyses with participants who responded ‘I do not know’ or ‘refuse to answer’ added to the LBG category, 66 patients (11.3%), 36 siblings (6.0%), and 23 controls (6.4%) were classified as LBG. The unadjusted OR for LBG status was 1.37 (95% CI 1.07–1.76; *p* = 0.012) for patients compared to controls. Age- and gender-adjusted OR was 1.42 (95% CI 1.09–1.85; *p* = 0.009).

## Discussion

### Main findings

This large population-based case–control study found that the prevalence of a sexual minority status was higher in patients with NAPD (6.8%) than in siblings (4.3%) and in healthy controls (2.8%). Whereas approximately 80% of controls had disclosed their sexual identity to almost everyone in their lives, only 30% of patients had done so. Our study results provide preliminary evidence that sexual minority status is a risk factor for NAPD with a positive significant association OR of 1.6. Mean scores of social adversity, with the exception of CT, were significantly higher in LBGs than in HTSs, also within the patient group. CT, a history of bullying, and perceived discrimination partially mediated the association between sexual minority status and NAPD.

### Comparison to previous studies and interpretation

Chakraborty et al. 2011 found elevated rates of psychotic disorders in non-HTS individuals, 3.75 (1.76–8.00) unadjusted OR (95% CI). Similarly, in the Netherlands, in another general population study, Gevonden et al. (2014) found elevated rates of psychotic symptoms in LBG population compared with HTS during two consecutive periods: NEMESIS-1 (OR 2.56, 95% CI 1.71–3.84) and NEMESIS-2 (OR 2.30, 95% CI 1.42–3.71). In the current clinical sample, an OR of LBG status of 1.6 for patients with NAPD (95% CI 1.13–2.29, *p* = 0.008). Correspondingly, perceived discrimination outcomes were higher for LBGs in both of the aforementioned studies and thought to act as a social stressor (or threat) toward the genesis of psychopathology. Our mediation results confirm these reports by finding similar factors mediating the association between LBG status and NAPD specifically. Our results are also consistent with previous health mediation risk findings (Bontempo & D'Augelli, 2002) of data from 9188 9th–12th grade students from Massachusetts and Vermont; of whom 315 were LBGs. They showed a combined effect of sexual minority status and (high) victimization to be consistently associated with higher levels of risk indices such as substance use or suicide attempts. In our data, bullying experiences were more prevalent amongst LBG than HTS subjects, and the indirect effect of bullying on NAPD risk was significant. Compared to CT and discrimination, however, the effect of bullying was smaller. The reason for this may be that in the aforementioned study, bullying was ascertained in real time; while our study participants (mean age at current assessment 32) were older and re-call error could have led to an underreporting of bullying. Furthermore, 16% of our sexual minority patients had not disclosed their sexual identity, which also may have contributed to lower bullying scores.

Sexual minority status is likely to represent environmental factors that increase the risk for psychotic symptoms and disorders. Current environmental theories of psychosis emphasize a central role for adversive experiences over the life course. Childhood adversities, in particular recurrent experiences of hostility and threat, have been consistently associated with increased risk for psychotic disorder (Morgan & Gayer-Anderson, 2016). Similarly, higher rates of psychosis in immigrants and their offspring are likely to be explained by a negative social minority position, being part of a group that is viewed as inferior by the majority population (Veling, 2013) and chronic stress due to social exclusion, discrimination, and social defeat (Selten, van der Ven, Rutten, & Cantor-Graae, 2013). Such experiences are common in LGB individuals, even if they have not disclosed their sexual identity, by identification with the minority group (Meyer, 2003). Indeed, aversive social experiences partially mediated the effect of LGB status on the risk for psychotic disorder in our sample. Several authors (Howes & Murray, 2014) hypothesize that exposure to social stressors during critical periods of brain development leads to sensitization, resulting in permanent excess of basal pre-synaptic transmission of dopamine, which is thought to increase the risk for psychosis. Pathogenic effects of social stressors on neurochemical systems are similar for both NAPD and LBGs (Mizrahi, 2016).

Sexual identity disclosure has been shown to improve the overall mental health of LBG youth (Meyer, 2003), and adult LBGs who have disclosed their sexual preference show lower cortisol levels and less psychiatric symptoms compared to LBGs who have not (Juster et al., 2013). It is plausible that these neurodevelopmental and biological mechanisms, if present, are more pronounced in LBGs considering the trying conditions under which LBGs become of age and live in thereafter. LBGs are known to achieve important milestones such as a steadfast identity, settling down with a partner and family planning later in life (Kertzner, 2001). In spite of the Netherland's renowned international ‘gay-friendly’ reputation, our results show that LBGs experience increased psychological strain during their life course by means of social adversity. The formation of biased cognitive schemas is more likely to occur after negative social experiences, and are exacerbated and perpetuated by having an ‘outsider status’ (Veling, 2013). On the other hand, self-disclosure at a young age, which appears to be a trend (Russell & Fish, 2016), may lead to increased social adversity and exclusion in individuals not yet psychologically equipped to handle the adversity. This in turn might explain why the prevalence of psychiatric disorder in young LBGs has not declined over recent decades, despite positive changes in social attitudes in Western countries (Brechwald, 2011). In addition to the above-mentioned socio-neurodevelopmental theories, other potential mediators of association between LBGs and psychotic disorders should also be considered, such as healthy identity and body-image formation. A recent Dutch survey study of LBGs (*n* **=** 2352) showed that in men higher levels of gender non-conformity predicted the experiences of CT by an adult family member, which in turn predicted the higher level of adult revictimization. If LBGs are more victimized as children by primary caregivers (Bos, de Haas, & Kuyper, 2019), they are also more likely to be deprived of the developmental conditions needed to form a stead-fast sense of self and a healthy body-image. Difficulties in establishing a stead-fast sense of self are reported by patients with psychosis (Nelson, Thompson, & Yung, 2013).

### Strengths and limitations

The results of this study should be interpreted in the light of several methodological issues. Selection bias may have occurred. While the large patient group of the GROUP study can be argued to be representative of the NAPD population (Korver et al., 2012), at the third assessment (6 years after baseline), 48% of the original patient sample (*n* = 1120) was lost to follow-up. The results would be biased if HTS patients were more likely to drop out than LBG patients, or if healthy LBGs were less likely to participate in the study than HTS controls. We tackled possible responder bias, by allocating ‘the refuse to answer’ and ‘I don't know’, a substantial total of 13 participants to the HTS group. A recent population survey found that approximately 4% and 3% of Dutch men and women, respectively, are homosexual (Keuzekamp, Kooiman, & Lisdonk, 2012), this corresponds well with the LBG rate in our control group. We conceptualized predominant same-sex attraction as a measure of sexual minority identity (i.e. the self-identification of LGB), yet we recognize that we did not also ask about same-sex behavior and predominant attraction, does not *per se* necessitate same-sex behavior or self-identification as an LBG individual. However, dissonance between sexual identity, in which case same-sex attraction is a key question to pose (Sell, 1997), and same-sex behavior occurs particularly in (young) adolescents (Kann et al., 2016), whereas the mean age in our minority patients was 34.9 years of age.

A further potential concern is the measurement error of sexual minority status. It is conceivable that sexual identity is a part of delusional ideas in some patients with NAPD. Sexual orientation was measured 6 years after baseline, making incorrect classification as LBG as a result of actual psychosis less likely.

Furthermore, mediators must precede the occurrence of the outcome in time. This is true for CT and bullying, but not necessarily for perceived discrimination, as this was measured lifetime and could, therefore, may have occurred after the onset of psychosis. Another limitation of the current study is the small LBG sample size. We did not have enough statistical power to control for urban living and cannabis use. LBGs tend to live in densely populated urban areas (Kuyper & Fokkema, 2011). Higher occurrence of substance abuse amongst LBGs is a well-replicated finding (Bos et al., 2019) and is by some hypothesized to be more ‘normalized’ within the LBG culture and/or used as a coping mechanism for minority stress (Meyer, 2003). It should be acknowledged that these variables had many missing values in third wave data, which limits their interpretation. Our data suggest that cannabis use was lower in LBGs than HTSs, which implies it is probably not a substantial factor in explaining the increased risk for psychosis in this population. As we did not have detailed information on cannabis use, and data were not available for a third of participants, conclusions should be regarded with caution. The results of this study implicate that LBGs have even more increased mental health risks, than previously known. Social defeat factors such as CT, discrimination, and bullying especially need to be addressed.

## References

[ref1] Andreasen, N. C., Flaum, M., & Arndt, S. (1992). The comprehensive assessment of symptoms and history (CASH). An instrument for assessing diagnosis and psychopathology. Archives of General Psychiatry, 49(8), 615–623. doi: 10.1001/archpsyc.1992.01820080023004. 1637251

[ref2] APA (2000). Diagnostic and statistical manual of mental disorders (4th ed.) (DSM-IV-TR), Washington, DC: American Psychiatric Association.

[ref3] Bebbington, P. E., Bhugra, D., Brugha, T., Singleton, N., Farrell, M., Jenkins, R., … Meltzer, H. (2004). Psychosis, victimisation and childhood disadvantage: Evidence from the second British national survey of psychiatric morbidity. The British Journal of Psychiatry, 185, 220–226. doi: 10.1192/bjp.185.3.220. 15339826

[ref4] Bontempo, D. E., & D'Augelli, A. R. (2002). Effects of at-school victimization and sexual orientation on lesbian, gay, or bisexual youths’ health risk behavior. The Journal of Adolescent Health, 30(5), 364–374. doi: 10.1016/s1054-139x(01)00415-3. 11996785

[ref5] Bos, H., de Haas, S., & Kuyper, L. (2019). Lesbian, gay, and bisexual adults: Childhood gender nonconformity, childhood trauma, and sexual victimization. Journal of Interpersonal Violence, 34(3), 496–515. doi: 10.1177/0886260516641285. 27036153

[ref6] Brechwald, W. A., & Prinstein, M. J. (2011). Beyond homophily: A decade of advances in understanding peer influence processes. Journal of Research on Adolescence, 21(1), 166–179. doi: 10.1111/j.1532-7795.2010.00721.x. 23730122 PMC3666937

[ref7] Chakraborty, A., McManus, S., Brugha, T. S., Bebbington, P., & King, M. (2011). Mental health of the non-heterosexual population of England. The British Journal of Psychiatry, 198(2), 143–148. doi: 10.1192/bjp.bp.110.082271. 21282785

[ref8] Cochran, S. D., & Mays, V. M. (2000). Relation between psychiatric syndromes and behaviorally defined sexual orientation in a sample of the US population. American Journal of Epidemiology, 151(5), 516–523. doi: 10.1093/oxfordjournals.aje.a010238. 10707921 PMC3698226

[ref9] Corliss, H. L., Cochran, S. D., & Mays, V. M. (2002). Reports of parental maltreatment during childhood in a United States population-based survey of homosexual, bisexual, and heterosexual adults. Child Abuse & Neglect, 26(11), 1165–1178. doi: 10.1016/s0145-2134(02)00385-x. 12398854 PMC4194076

[ref10] Gevonden, M. J., Selten, J. P., Myin-Germeys, I., de Graaf, R., ten Have, M., van Dorsselaer, S., … Veling, W. (2014). Sexual minority status and psychotic symptoms: Findings from the Netherlands mental health survey and incidence studies (NEMESIS). Psychological Medicine, 44(2), 421–433. doi: 10.1017/S0033291713000718. 23710972

[ref11] Goodenow, C., Watson, R. J., Adjei, J., Homma, Y., & Saewyc, E. (2016). Sexual orientation trends and disparities in school bullying and violence-related experiences, 1999-2013. Psychology of Sexual Orientation and Gender Diversity, 3(4), 386–396. doi: 10.1037/sgd0000188. 29322064 PMC5758340

[ref34] Hayes, A. F. (2012). PROCESS: A versatile computational tool for observed variable mediation, moderation, and conditional process modeling [White paper]. Retrieved from http://www.afhayes.com/public/process2012.pdf.

[ref12] Howes, O. D., & Murray, R. M. (2014). Schizophrenia: An integrated sociodevelopmental-cognitive model. Lancet *(*London, England*)*, 383(9929), 1677–1687. doi: 10.1016/S0140-6736(13)62036-X. PMC4127444 24315522

[ref13] Juster, R. P., Smith, N. G., Ouellet, E., Sindi, S., & Lupien, S. J. (2013). Sexual orientation and disclosure in relation to psychiatric symptoms, diurnal cortisol, and allostatic load. Psychosomatic Medicine, 75(2), 103–116. doi: 10.1097/PSY.0b013e3182826881. 23362500

[ref14] Kann, L., Olsen, E. O., McManus, T., Harris, W. A., Shanklin, S. L., Flint, K. H., … Zaza, S. (2016). Sexual identity, sex of sexual contacts, and health-related behaviors among students in grades 9–12 – United States and selected sites, 2015. Morbidity and Mortality Weekly Report. *Surveillance Summaries* (*Washington, D.C.:* 2002), 65(9), 1-202. doi: 10.15585/mmwr.ss6509a1. 27513843

[ref15] Kertzner, R. M. (2001). The adult life course and homosexual identity in midlife gay men. Annual Review of Sex Research, 12, 75–92. 12666737

[ref16] Keuzekamp, S., Kooiman, N., & Lisdonk, J. (2012). *Niet te ver uit de kast* (Report No. 2012-10). Den Haag: Sociaal Cultureel Planbureau. Retrieved from https://www.scp.nl/Publicaties/Alle_publicaties/Publicaties_2012/Niet_te_ver_uit_de_kast.

[ref17] King, M., Semlyen, J., Tai, S. S., Killaspy, H., Osborn, D., Popelyuk, D., & Nazareth, I. (2008). A systematic review of mental disorder, suicide, and deliberate self harm in lesbian, gay and bisexual people. BMC Psychiatry 8, 70. doi: 10.1186/1471-244X-8-70. 18706118 PMC2533652

[ref18] Korver, N., Quee, P. J., Boos, H. B., Simons, C. J., & de Haan, L., & GROUP investigators. (2012). Genetic risk and outcome of psychosis (GROUP), a multi-site longitudinal cohort study focused on gene-environment interaction: Objectives, sample characteristics, recruitment and assessment methods. International Journal of Methods in Psychiatric Research, 21(3), 205–221. doi: 10.1002/mpr.1352. 22419500 PMC6878383

[ref19] Kuyper, L., & Fokkema, T. (2011). Minority stress and mental health among Dutch LGBs: Examination of differences between sex and sexual orientation. Journal of Counseling Psychology, 58(2), 222–233. doi: 10.1037/a0022688. 21401219

[ref20] Lataster, T., van Os, J., Drukker, M., Henquet, C., Feron, F., Gunther, N., (2006). Childhood victimisation and developmental expression of non-clinical delusional ideation and hallucinatory experiences: Victimisation and non-clinical psychotic experiences. Social Psychiatry and Psychiatric Epidemiology, 41(6), 423–428. doi: 10.1007/s00127-006-0060-4. 16572272

[ref21] Meyer, I. H. (2003). Prejudice, social stress, and mental health in lesbian, gay, and bisexual populations: Conceptual issues and research evidence. Psychological Bulletin, 129(5), 674–697. doi: 10.1037/0033-2909.129.5.674. 12956539 PMC2072932

[ref22] Mizrahi, R. (2016). Social stress and psychosis risk: Common neurochemical substrates? Neuropsychopharmacology, 41(3), 666–674. doi: 10.1038/npp.2015.274. 26346639 PMC4707841

[ref23] Morgan, C., & Gayer-Anderson, C. (2016). Childhood adversities and psychosis: Evidence, challenges, implications. World Psychiatry, 15(2), 93–102. doi: 10.1002/wps.20330. 27265690 PMC4911761

[ref24] Nelson, B., Thompson, A., & Yung, A. R. (2013). Not all first-episode psychosis is the same: Preliminary evidence of greater basic self-disturbance in schizophrenia spectrum cases. Early Intervention in Psychiatry, 7(2), 200–204. doi: 10.1111/j.1751-7893.2012.00381.x. 22759705

[ref25] Pearce, J., Rafiq, S., Simpson, J., & Varese, F. (2019). Perceived discrimination and psychosis: A systematic review of the literature. Social Psychiatry and Psychiatric Epidemiology, 54(9), 1023–1044. doi: 10.1007/s00127-019-01729-3. 31236631

[ref27] Russell, S. T., & Fish, J. N. (2016). Mental health in lesbian, gay, bisexual, and transgender (LGBT) youth. Annual Review of Clinical Psychology, 12, 465–487. doi: 10.1146/annurev-clinpsy-021815-093153. PMC4887282 26772206

[ref28] Sell, R. L. (1997). Defining and measuring sexual orientation: A review. Archives of Sexual Behavior, 26(6), 643–658. doi: 10.1023/a:1024528427013. 9415799

[ref29] Selten, J. P., van der Ven, E., Rutten, B. P., & Cantor-Graae, E. (2013). The social defeat hypothesis of schizophrenia: An update. Schizophrenia Bulletin, 39(6), 1180–1186. doi: 10.1093/schbul/sbt134. 24062592 PMC3796093

[ref30] Thombs, B. D., Bernstein, D. P., Lobbestael, J., & Arntz, A. (2009). A validation study of the Dutch childhood trauma questionnaire-short form: Factor structure, reliability, and known-groups validity. Child Abuse & Neglect, 33(8), 518–523. doi: 10.1016/j.chiabu.2009.03.001. 19758699

[ref31] van Nierop, M., Lataster, T., Smeets, F., Gunther, N., van Zelst, C., de Graaf, R., … van Winkel, R. (2014). Psychopathological mechanisms linking childhood traumatic experiences to risk of psychotic symptoms: Analysis of a large, representative population-based sample. Schizophrenia Bulletin, 40 (Suppl 2), S123–S130. doi: 10.1093/schbul/sbt150. 24562491 PMC3934395

[ref32] Veling, W. (2013). Ethnic minority position and risk for psychotic disorders. Current Opinion in Psychiatry, 26(2), 166–171. doi: 10.1097/YCO.0b013e32835d9e43. 23286992

[ref33] Wing, J. K., Babor, T., Brugha, T., Burke, J., Cooper, J. E., Giel, R., … Sartorius, N. (1990). SCAN. Schedules for clinical assessment in neuropsychiatry. Archives of General Psychiatry, 47(6), 589–593. doi: 10.1001/archpsyc.1990.01810180089012. 2190539

